# Peak systolic velocity using color-coded tissue Doppler imaging, a strong and independent predictor of outcome in acute coronary syndrome patients

**DOI:** 10.1186/1476-7120-11-9

**Published:** 2013-04-01

**Authors:** Carl Westholm, Jonas Johnson, Anders Sahlen, Reidar Winter, Tomas Jernberg

**Affiliations:** 1 Department of Medicine, Section of Cardiology, Karolinska Institutet, Stockholm, Sweden; 2 Departement of Cardiology, Karolinska University Hospital, Stockholm, Sweden; 3 School of Technology and Health, Royal Institute of Technology, Stockholm, Sweden

**Keywords:** Acute coronary syndrome, Prognostic parameters, Tissue Doppler, Peak systolic velocity

## Abstract

**Background:**

Traditional echocardiographic methods like left ventricular ejection fraction(EF) and wall motion scoring (WMS) and new methods like speckle tracking (ST) based 2D strain carry important prognostic information in acute coronary syndrome (ACS) patients. Parameters from tissue Doppler imaging (TDI), with its high time resolution, may further increase the prognostic value. Peak systolic velocity (PSV) of the basal segments of the left ventricle from TDI is a robust and user independent parameter. The aim was to investigate the prognostic value of PSV compared to EF, WMS, 2D strain and E/e'.

**Methods:**

Echocardiographic images were collected and post processed in 227 ACS patients. Additional clinical data was prospectively gathered and patients were followed for 3-5 years regarding the combined endpoint of death or re-admission due to ACS or heart failure.

**Results:**

The combined endpoint occurred in 85 (37%) patients. Those with an event had lower median PSV than those without (4,4 cm/s) vs. (5,3 cm/s), (p<0.001). In a ROC analysis, the AUC was larger for PSV (0.75) than for EF (0.68), WMS (0.63), 2D strain (0.67) and E/e'(0.70). The combined endpoint increased with decreasing PSV. When adjusting for differences in baseline characteristics in a COX-regression model, PSV remained independently associated with outcome where the others did not. PSV was also less sensitive to image quality with fewer values missing or unacceptable for analysis.

**Conclusion:**

Peak systolic velocity (PSV) is a robust measurement that seems to have a strong and independent association with outcome compared to traditional echocardiographic measurements in ACS patients.

## Background

Patients admitted to hospital because of an acute coronary syndrome (ACS) constitute a heterogeneous population with a varying risk of future cardiac events. To identify high-risk patients who may benefit from an intensified treatment strategy and low-risk patients who may benefit from a more conservative approach and early discharge, risk stratification is important. Traditionally, echocardiographic measurements of left ventricular systolic function, such as ejection fraction (EF) and wall motion scoring (WMS), have been considered as essential in this process [1,2] However both EF and WMS are limited by being highly user dependent with a rather long learning curve and poor reproducibility [3,4]. Subsequent studies have introduced the diastolic echocardiographic parameter E/e' -ratio, that reflects the filling pressure of the left ventricle [5] as a prognostic parameter with incremental value to EF and WMS [6,7]. It has also been shown that this parameter adds complementary prognostic value to biochemical markers such as type B natriuretic peptide [8,9] after acute myocardial infarction (AMI) [10,11].

A more recently developed quantitative parameter like strain obtained from two-dimensional (2D) echocardiography based speckle tracking or tissue doppler imaging (TDI) has also been shown to add prognostic value to the traditional parameters [12,13]. This measurement derived from speckle tracking or TDI is less user dependent and more easily accessible compared to 2D-derived EF, WMS and E/e' although problems with feasibility remains [14-17].

Whereas systolic values of parameters like global systolic strain and the diastolic parameter E/e'-ratio have been shown useful for risk stratification in ACS patients, less is known about the prognostic value of systolic tissue velocities in this group of patients.

**Figure 1:**
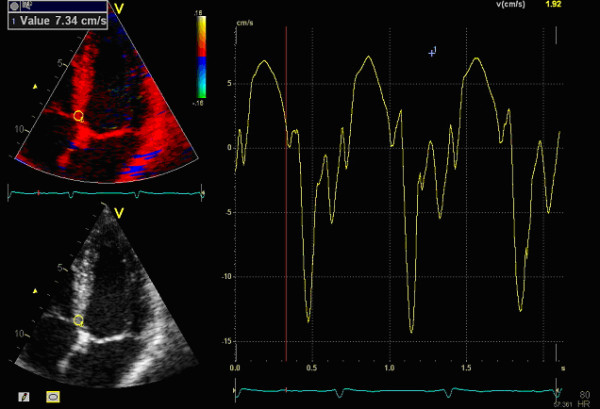
**How the peak systolic velocity (PSV) information is acquired.** The region of interest (ROI) is manually placed in the basal segments of the left ventricle and the corresponding tissue velocity is showed in the graph to the right where you can register the PSV guided by the ECG below in the picture. The time is on the x-axis and velocity in cm/s on the y-axis.

Peak systolic velocity (PSV) of the basal segments of the left ventricle from TDI is a feasible and relatively user independent measure (Figure 1). Tissue Doppler imaging has a higher time resolution than 2D-speckle tracking echocardiography and can therefore register processes not visible for the human eye or for speckle tracking [18,19]. The aim of this study was to investigate the prognostic value and feasibility of PSV compared to both traditional parameters, such as EF, WMS, E/e'-ratio and more recent such as 2D-strain.

## Method

### Study population

The study included patients admitted to the coronary care unit at Karolinska University Hospital, Huddinge, between August 2006 and January 2008 with a clinical diagnosis of ACS including unstable angina pectoris, ST elevation myocardial infarction and non ST elevation myocardial infarction. The patients were consecutively included except for temporary interruptions of the study due to high work load at the coronary care unit. Cardiac comorbidities such as valvular disease or arrhythmia were not reasons for exclusion. All patients underwent clinical assessment including clinical history, physical examination, standard 12-lead ECG, ECG-monitoring and serial measurement of biochemical cardiac markers up to 9–12 hours after admission. All other examinations and treatments were left to the discretion of the individual cardiologist. Clinical data were prospectively collected and entered into a database. An acute MI was defined according to current guidelines [20].

The combined end point was death from any cause, MI and rehospitalisation for heart failure. All in-hospital events were registered in the study database. Only new MIs occurring more than 24 hours after admission were considered as events. Out of hospital, information about death and need for readmission because of MI or heart failure was obtained by merging the database with the National Death Registry, which includes information of the vital status of all Swedish citizens, and the National Patient Registry, which includes diagnoses on all patients hospitalized in Sweden.

All patients received oral and written information about the study and written informed consent was obtained before entering the study. The study was conducted according to the principles of the Declaration of Helsinki and was approved by the local ethics committee.

### Laboratory analyses

N-terminal pro B-type natriuretic peptide (NT-proBNP) was determined 24 hours after admission by using the assay from Roche Diagnostics on a Modular Analytics E170. The analytical range extends from 20 to 35000 ng/L. Plasma creatinine was analysed on admission. Estimated glomerular filtration rate (eGFR) was calculated by using the Cockcroft-Gault formula [21].

### Echocardiographic acquisition and analyses

All echocardiography data was collected during the first days (median (25th-75th percentile): 3(2–4)) of admission according to the local standard clinical protocol at that time on Karolinska University Hospital by the cardiologist or cardiac technician on duty that day. The images, including 2D, TDI and spectral Doppler, were collected with a GE Vingmed vivid7 ultrasound machine with a M3S or M4S transducer and standard installed software.

The images were analyzed using a dedicated workstation (EchoPAC, GE Healthcare, Horten Norway) by a well-trained cardiologist (CW) blinded to baseline data and subsequent outcome.

Ejection fraction was measured according to current EAE/ASE recommendations [22] using the biplane Simpson method of discs from outlining the endocardial border in the apical 4-, and 2-chamber views.

Wall motion score was determined as the mean value of an 18 segment model from the three apical views, where each segment was given a score of 1–3, in which 1 represented normokinesia, 2 indicated hypokinesia, and 3, akinesia.

Two dimensional-strain was derived from apical 2D images using the EchoPAC software. Global 2D strain was calculated as the mean of the regional longitudinal strain of the 18 different segments and was calculated if the strain was measurable in at least 12 segments. The Automatic tracking was visually controlled and manually corrected if needed. Strain information can also be obtained from TDI but in this material the apical projections of TDI did often not include the apical segments and quite often also just parts of the midventricular segment and therefore we concluded that Strain from TDI could not be obtained in this study.

E/e' was calculated from the E-wave of the mitral inflow to the LV measured by pulsed Doppler and e' was measured from the four chamber TDI image as the mean of the septal and lateral maximum diastolic velocities.

PSV was measured from TDI images, as illustrated in Figure 1. The global PSV value was defined as the mean of the velocities from the 6 different basal segments of the left ventricle. We excluded velocity peaks from the isovolumetric phases with end systole defined by the closing of the aortic valve in the three chamber projection. As mentioned above, the images were collected by various sonographers and physicians during the inclusion without standardized frame rate which therefore varied between 100 and 150 frames/s between patients. As the longitudinal velocities are a more global parameter compared to Strain with co variation between adjacent segments we calculated PSV if the basal velocity was measureable in at least 2 of 6 segments. To confirm that assumption the correlation between the PSV only from the septal and lateral wall and that of PSV as mean from all basal segments was examined.

Image quality was registered using a four-grade scale, as well as the reason for a missing value (non existing image or too poor quality to allow analysis).

To investigate the interobserver variability, another well-trained cardiologist (AS) measured PSV on 20 randomly chosen patients. The variability was evaluated by calculating the Coefficient of Variation (CV) using the formula “CV = Standard deviation / expected return” where the expected return is the mean of all the measurements by both readers, and the standard deviation is of the differences between the paired readings. As PSV was our main focus in this study we did not investigate the CV of other parameters that has already been investigated [16].

### Statistical analysis

Continuous data are presented as medians with interquartile range (IQR) and categorical data are presented with frequencies and percent. When analyzing differences between groups the Mann Whitney-test was used for continuous variables and Chi2-test for categorical variables. To compare the prognostic value regardless of chosen cut off-value, we used receiver operating characteristics (ROC) analyses expressing prognostic value as area under curve (AUC) with 95% confidence interval (CI) and significance tested according to Hanley and McNeil [23] To illustrate the timing of events, Kaplan Meier curves were generated. Correlations were evaluated with Spearman’s rho.

To identify which echocardiographic parameters were independent predictors of outcome we used Cox-regression analyses, adjusting for other variable well known to be associated with outcome (age, gender, diabetes, hypertension and previous heart failure) in three different models. In model 1 each tested echocardiographic parameter was entered one by one together with the above mentioned risk factors. In model 2 we also included NT proBNP and eGFR among the risk factors to adjust for. In model 3 we removed NT proBNP and eGFR, and instead inserted PSV among the risk factors to adjust for. To evaluate whether early intervention influenced the association between PSV and outcome, coronary angiography and treatment with PCI or CABG were also included in the models.

## Results

A total of 227 patients were included in the study. The median follow up time was 53 (48–58) months. During this period 85 (37%) patients reached the combined endpoint, among them 42 (19%) died, 48 (21%) had a MI and 52 (23%) were readmitted because of heart failure. Baseline characteristics, laboratory data and final diagnoses at the index event in all patients and in those with and without a subsequent event are listed in Table 1. Patients with an event were older and had more often hypertension and a previous history of heart failure. They also had a higher level of NT-proBNP and a lower eGFR. Coronary angiography was performed in 192 patients, of which 109 and 25 underwent percutaneous coronary intervention (PCI) and coronary bypass surgery (CABG), respectively.

**Table 1:** Baseline characteristics (n = 227)

	**All**		**No death, MI or HF**		**Death, MI or HF**		
	**(n=227)**		**(n= 142)**		**(n=85)**		
**Variables**	**n**	**(%)**	**n**	**(%)**	**n**	**(%)**	**p**
**Demographics:**							
Age (median, 25th-75th perc.)	67	(59–77)	62	(56–74)	74	(63–80)	<0,001
Men	172	(76)	111	(78)	61	(72)	0.276
**Risk factors:**							
Hypertension	121	(53)	63	(44)	58	(68)	<0.001
Diabetes Mellitus	51	(22)	29	(20)	22	(26)	0.340
Current smoker (missing n=6)	43	(19)	33	(23)	10	(13)	0.049
**Previous cardiovascular disease:**							
Myocardial infarction	56	(25)	31	(22)	25	(30)	0.200
Heart Failure	19	(8)	2	(1)	17	(20)	<0.001
Revascularization, PCI	33	(15)	23	(16)	10	(12)	0.359
Revascularization, CABG	10	(4)	4	(3)	6	(7)	0.132
Stroke	17	(7)	10	(7)	7	(8)	0.741
**Laboratory measurements**							
NTproBNP 24 h (median, 25th-75th perc.) (n=189))			724	(303–1887)	2300	(1030–26040)	<0.001
eGFR (median, 25th-75th perc.) (n=220)			92	(72–116)	66	(40–96)	<0.001
**Index Diagnosis**							
Myocardial infarction	188	(83)	119	(84)	69	(81)	0.612

MI=Myocardial infarction, HF=Heart failure, eGFR=glomerular filtration rate.

### Echocardiographic acquisition and measurements

Peak systolic velocity could be measured in all except 3 (1%) patients, whereas EF Simpson and WMS could not be assessed in 19 (8%) and 9 (4%) patients, respectively. 2D-strain could not be obtained in 41 (18%) of the patients and the corresponding number for E/e' was 28 (12%). The results of the echocardiographic measurements are listed in Table 2. When assessing the interobserver variability between two observers regarding PSV measurements the CV was 5.9%. The correlation (Spearman’s rho) between global PSV calculated from 6 basal segments and PSV calculated from only the septal and lateral segment was 0.91.

**Table 2:** Echocardiographic measurements

	**All**		**No death, MI or HF**		**Death, MI or HF**		
	**(n=227)**		**(n= 142)**		**(n=85)**		
**Variables**	**median**	**(25th-75th perc)**	**median**	**(25th-75th perc)**	**median**	**(25th-75th perc)**	**p**
PSV	4.9	(4.2-5.8)	5.3	(4.7-6.0)	4.4	(3.6-5.0)	<0.001
EF	49	(41–56)	52	(45–58)	45	(35–52)	<0.001
WMS	1.0	(1.0-1.3)	1.26	(1.09-1.16)	1.29	(1.21-1.36)	<0.001
Global 2D-Strain	−14	(−18- -9.8)	−15	(−19- -11)	−11	(−16.8- -5.0)	<0.001
E/e'-ratio	13.4	(10.1-17.6)	13.2	(12.3-14.2)	18.4	(16.1-20.8)	<0.001

MI=Myocardial infarction, HF=Heart failure, PSV=peak systolic velocity, EF=ejection fraction, WMS=Wall motion scoring.

### Prognostic value of peak systolic velocity in comparison to other echocardiographic data

**Figure 2:**
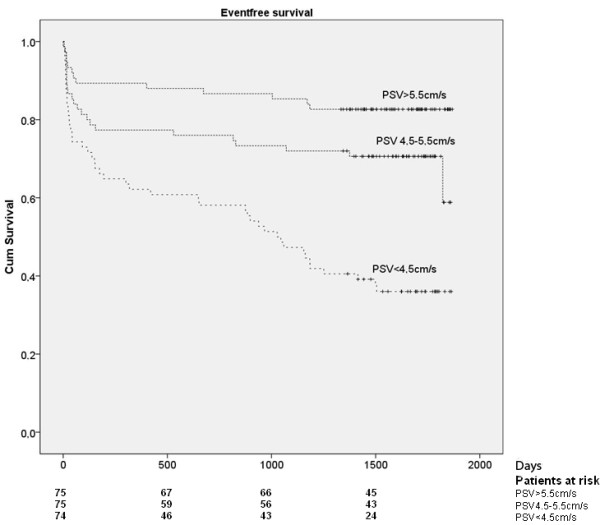
Combined endpoint (death, myocardial infarction or readmission because of heart failure) in relation to PSV measurement.

Patients with an combined event had lower median PSV than those without (4,4 cm/s) vs. (5,3 cm/s), (p<0.001). These groups also differed significantly regarding EF Simpson, WMS, strain and E/e'-ratio (Table 2). In ROC analyses, the AUC (Area under curve) was larger for PSV than for the other parameters but the differences did not reach statistical significance (Table 3). When patients were divided into tertiles according to the PSV measurements the long term risk of subsequent cardiac events increased with decreasing PSV (Figure 2). The association between PSV and outcome was apparent for all individual endpoints (Table 4).

**Table 3:** Area under curve according to ROC-analysis

**Variables**	**AUC (95%CI)**
PSV	0.75 (0.68-0.81)
EF	0.68 (0.61-0.76)
WMS	0.64 (0.56-0.72)
Global 2D-Strain	0.67 (0.58-0.75)
E/e'-ratio	0.70 (0.62-0.77)

**Table 4:** Events according to different PSV:s

	**PSV <4.5 cm/s**	**PSV 4.5-5.5 cm/s**	**PSV >5.5 cm/s**
Death	26	12	4
Re-admission due to heart failure	32	15	4
New MI	26	14	8
Combined endpoint (all the above)	47	24	13

### Independent predictors of outcome

All tested echocardiographic parameters except 2D-strain remained independently associated with outcome when these variables one at the time were adjusted for age, gender, diabetes, hypertension and previous heart failure (Table 5, model 1). When patients in the third tertile according to PSV (>5.5 cm/s) were used as reference the HR(95%CI) increased to 1.65 (0.80-3.37) p=0.169, and 3.16 (1.58-6.35) p=0.001 in the second and first tertile, respectively. In model 2 where NT-proBNP and eGFR were added to the model, only PSV, and E/e' and remained independent predictors of outcome (Table 5, model 2). When including PSV in the model, PSV was the only echocardiographic parameter independently associated with outcome (Table 5, model 3).

**Table 5:** Cox-regression analysis

**Parameter**	**Model 1.**		**Model 2.**		**Model 3.**	
	**Adjusted for age, gender, diabetes, hypertension and previous heart failure.**		**Adjusted for the same factors as model 1 plus NT proBNP 24 h and eGFR**		**Adjusted for the same as model 1 plus PSV. The corresponding results for PSV is also presented for each parameter HR (95%CI)**	
	**HR (95%CI)**	**p**	**HR (95%CI)**	**p**	**HR (95%CI)**	**p**
PSV	0.65 (0.51-0.82)	<0.001	0.71 (0.54-0.93)	0.013	-------------	
					-------------	
EF	0.97 (0.95-0.99)	0.006	0.98 (0.96-1.00)	0.070	0.99 (0.96-1.01)	0.227
					**PSV** 0.68 (0.52-0.90)	0.007
WMS	2.09 (1.06-4.15)	0.035	1.52 (0.71-3.24)	0.280	0.95 (0.41-2.22)	0.915
					**PSV** 0.61 (0.46-0.82)	0.001
Global 2D Strain	1.03 (0.99-1.07)	0.003	1.04 (0.96-1.05)	0.037	1.02 (0.98-1.06)	0.249
					**PSV** 0.67 (0.50-0.90)	0.032
E/e'	17.97 (2.24-144.01)	0.007	20.0 (2.31-174.4)	0.007	6.90 (0.740-64.27)	0.090
					**PSV** 0.70 (0.54-0.91)	0.008

When we included intervention with PCI or CABG in the model, PSV remained significantly associated with outcome, (HR(95%) 0.66 (0.51-0,84), p=0.001).

## Discussion

This study demonstrates that PSV can be measured in most patients with a low interobserver variability and may even be a better predictor of outcome than both traditional echocardiographic measurements of left ventricular function, such as EF, WMS and E/e'-ratio and more recent deformation parameters, such as myocardial strain in ACS patients.

The aim of this study was to test PSV in a real world setting. We therefore included consecutive unselected ACS patients and all images were acquired as in the clinical routine. In the univariable analyses, the median PSV value was lower in those with than in those without a subsequent cardiac event. When categorizing patients according PSV values, a low-, intermediate- and high-risk group could be identified with a 5-year cumulative risk of death, MI or readmission because of heart failure of 17%, 32% and 64%, respectively. Even after adjusting for differences in well-known predictors of outcome (including natriuretic peptides and estimates of kidney function), there was a strong association between PSV and outcome, with a 1.4 fold increased risk of the combined endpoint for every unit decrease in PSV.

When the prognostic value of PSV was compared with that of other echocardiographic parameters in a ROC analysis, PSV had the largest AUC although the differences were not statistical significant. When the echocardiographic parameters were tested one by one in a regression model including well-known predictor of outcome, only PSV and E/e'-ratio remained associated with outcome, whereas EF, WMS and strain failed to independently predict outcome. Finally, when PSV was included in the model, none of the other echocardiographic parameters carried any additional prognostic information.

The predictive value of different echocardiographic parameters may vary in different patient populations. Wang et al. [6] demonstrated a better prognostic value for both é, PSV and E/e' than for EF and WMS. In that study e' was the strongest predictor of cardiac death although PSV and E/e' also were strong predictors of cardiac death. However, the study by Wang and co-workers included patients with a broad spectrum of heart disease and only 16% had ischemic heart disease. The fact that the diastolic parameter e' was demonstrated to have a higher prognostic value than PSV might be explained by a relatively small proportion of patients having ischemic heart disease and a larger proportion of patients having heart failure. In such a population the proportion of patients with high filling pressure is expected to be higher than in our study population, which included ACS patients with predominantly normal or only mildly depressed EF.

Our study does not only confirm but also extends previous findings showing that E/e' has an incremental prognostic value to that of natriuretic peptides [8,9]. Somewhat unexpectedly 2D-strain was not better to predict outcome than traditional measurements of systolic LV function such as EF and WMS. This is in contrast to earlier studies in the field. In a cohort of 649 ACS patients Antoni and co-workers showed that both strain and strain rate were superior to the traditional parameters, EF and WMS, to predict 1-year outcome [16] and similar finding were made by Bertoni and co-workers but in a population with chronic ischemic heart disease [24]. In our study, we could not confirm a superiority of deformation parameters in comparison to EF and WMS. Clearly, the operator’s skills and experience, and their compliance to strict protocols are important determinants of the quality of echocardiographic examinations. It is important to note that the images in our study was collected from routine clinical echocardiography not always of highest quality, but still sufficient for analyzing PSV in a very large proportion of the patients, whereas in the study of Antoni et al. and Bertoni et al., all exams were performed within a dedicated laboratory with great experience of conducting studies and using deformation analysis. Still, even if a technique is good in the hands of experts in specialized centers, it can be problematic in the more generalized everyday clinical situation if the demand for technical skill is too high [25].

Therefore, the simplicity of PSV must be regarded as a great advantage, which can be an important determinant to why PSV in our study is superior to the other parameters. Another advantage is the fact that the method seems robust and insensitive to poor image quality with a low interobserver variability and low number of missing values. Thus the results of this study indicates that PSV could be superior to other parameters as a clinical routine method outside a core laboratory to predict outcome. Furthermore the strong correlation (0.91) between global PSV from all 6 basal segments and PSV from only septal and lateral wall indicates that the use of only one projection and two measurement might be as predictive as the global PSV which would further simplifiy the method.

The present study has some limitations. The sample size was rather small. Thus, lack of significant differences may still be caused by lack of power to detect such differences and our findings need to be confirmed in larger studies. Although interobserver variability was assessed for PSV in a subgroup of patients and all analyses were performed according to a protocol, only one person performed the echocardiographic analyses. Due to the clinical setting and image quality, strain rate was not included in this study. Strain rate is the derivate of strain and therefore it is reasonable to believe that the variability and number of missing values would be even higher compared to strain.

This study used a prespecified combined endpoint. Although PSV was significantly associated with all individual endpoints, the underlying mechanisms for these associations may differ. Systolic LV-dysfunction is known to cause both heart failure and death, whereas the association between systolic LV-dysfunction and subsequent risk of new MI may be explained by the association between systolic LV-dysfunction and severity of coronary artery disease.

In conclusion, PSV seems to be a robust and easily obtained echocardiographic measurement that may be very useful for risk stratifying ACS patients.

## Competing interests

The authors declare that they have no competing interests.

## Authors’ contributions

All authors read and approved the final manuscript.
